# Effects of dose heterogeneity on response and toxicity after ^90^Y-SIRT for HCC

**DOI:** 10.1007/s00259-026-08064-1

**Published:** 2026-07-14

**Authors:** Huan Xi, Xinlin Zheng, Andor W.J.M. Glaudemans, Joyce van Sluis, Simeon J.S. Ruiter, Robbert J. de Haas, G. Matthijs Kater, Oleksandra V. Ivashchenko, Walter Noordzij

**Affiliations:** 1 Department of Nuclear Medicine and Molecular Imaging, University of Groningen, University Medical Centre Groningen, Groningen, The Netherlands; 2 Department of Hepato-Pancreato-Biliary Surgery and Liver Transplantation, University of Groningen, University Medical Centre Groningen, Groningen, The Netherlands; 3 Department of Radiology, University of Groningen, University Medical Centre Groningen, Groningen, The Netherlands

**Keywords:** Yttrium-90, Selective internal radiation therapy, Voxel-based dosimetry, Dose heterogeneity, Hepatocellular carcinoma

## Abstract

**Purpose:**

Yttrium-90 (^90^Y) selective internal radiation therapy (SIRT) is an established treatment for hepatocellular carcinoma (HCC), yet dose-response relationships remain incompletely understood. We evaluated whether voxel-based dose heterogeneity is associated with tumour response and treatment-related toxicity.

**Methods:**

This retrospective study included 45 HCC patients with 68 lesions treated with ^90^Y resin SIRT. Dose distributions were derived from post-therapy ^90^Y PET/CT. DVH-based metrics included hot-spot (D2, D10, Dmax), cold-spot (D90, D98, Dmin), and coverage parameters (V100, V150, V200). Tumour response was assessed using mRECIST. Grade ≥ 3 toxicity was classified as acute or latent. Lesions were stratified by median absorbed dose (D50 < 128 Gy vs. ≥ 128 Gy).

**Results:**

Of 68 lesions, 44% achieved complete response. D50 did not differ significantly across mRECIST response categories. In low-D50 lesions, higher heterogeneity was associated with improved response. Hot-spot metrics (D2, D10, Dmax) showed similar trends. In high-D50 lesions, cold-spot metrics (D90, D98) were significantly associated with better response. Mean non-tumoral liver dose was associated with acute toxicity, while dose heterogeneity was associated with latent toxicity.

**Conclusion:**

Spatial dose distribution influences outcomes beyond mean absorbed dose. Tumour response was related to a dose level dependent pattern, with hot-spot–driven heterogeneity associated with response at lower dose levels and cold-spot metrics more relevant at higher dose levels. In addition, a temporal dissociation in toxicity was identified, with mean non-tumoral liver dose associated with acute injury and spatial dose heterogeneity associated with latent severe toxicity, supporting further evaluation of spatial dosimetry for individualized SIRT planning.

**Supplementary Information:**

The online version contains supplementary material available at https://doi.org/10.1007/s00259-026-08064-1.

## Introduction

Yttrium-90 (^90^Y) Selective Internal Radiation Therapy (SIRT) is a well-established treatment for hepatocellular carcinoma (HCC), with effectiveness dependent on optimizing tumour dose delivery and minimizing non-tumoral liver deposition [1]. Tumour dose-response and dose-toxicity thresholds have been reported [2, 3]. However, current individual dosimetry focuses on mean dose, assuming the distribution of microspheres is uniform. In practice, post-therapy ^90^Y PET/CT imaging shows substantial spatial heterogeneity, reflecting complex microsphere distribution patterns influenced by tumour vascularity and perfusion dynamics [4].

The clinical implications of dose heterogeneity in ^90^Y-SIRT remain unclear. Heterogeneous microsphere distribution creates under-dosed tumour regions [5, 6], and higher minimum dose coverage has been associated with improved treatment response, suggesting that cold spots may contribute to treatment failure [7, 8]. However, dose heterogeneity is an inherent feature of SIRT, and emerging evidence from other radiation modalities suggests that heterogeneous dose distributions may activate multiple dose-dependent biological mechanisms within the tumour microenvironment [9]. Whether dose heterogeneity is detrimental to outcomes or may have therapeutic value has not been systematically evaluated using voxel-based dosimetry from post-therapy ^90^Y PET/CT. Beyond tumour response, the contribution of dose heterogeneity to toxicity has received limited attention. While mean non-tumoral liver dose (NTLD) has been associated with treatment-related toxicity [3], the impact of non-tumoral liver dose heterogeneity on toxicity risk remains unclear.

We therefore performed voxel-based dosimetry of post-therapy ^90^Y PET/CT to explore associations between dose heterogeneity and both tumour response and toxicity in HCC patients treated with resin microsphere SIRT, with stratification by median tumour dose to account for potential effect modification.

## Materials and methods

We conducted a retrospective analysis of HCC patients treated with ^90^Y resin microsphere SIRT at the University Medical Centre Groningen from October 2021 to January 2025. Lesions were included if they had a contrast-enhanced CT/MRI within 12 weeks before therapy, a tumour diameter ≥ 20 mm, and a follow-up contrast-enhanced CT/MRI at approximately 4 months. The study protocol was reviewed and approved by the Central ethics Review Board for non-WMO studies of the University Medical Centre Groningen (CTc UMCG, registration number 22218) and was conducted in accordance with the Declaration of Helsinki. The requirement for written informed consent was waived. Age, sex, history, subsequent treatment, and outcomes were extracted from the electronic patient data system (EPD).

### Dosimetry

Administered activity was determined using the partition model in most patients (*n* = 30) and MIRD-based planning for radiation segmentectomy (*n* = 15).

Post-therapy dosimetry was based on post-therapy ^90^Y PET/CT scans. Imaging was performed at a median delay of 20.63 h (range, 1.03–23.45) after SIRT. Post-therapy ⁹⁰Y PET/CT scans were acquired on Siemens Biograph Vision and Biograph Vision Quadra PET/CT systems with a PET emission acquisition time of 900 s. Images were reconstructed using a 3D (TOF) OSEM protocol (4 iterations, 5 subsets) with PSF modelling and a 7-mm Gaussian filter, based on a prior ^90^Y PET/CT phantom optimisation study evaluating quantification accuracy and partial-volume effects [10]. The resulting image matrix was 220 × 220 with a voxel size of 3.2 × 3.2 × 1.0 mm. Reconstructed images were quantitative activity concentration maps (kBq/mL), requiring no additional calibration within the dosimetry software.

Dosimetry was performed in MIM SurePlan (v7.0.4, MIM Software, Cleveland, USA). A nuclear medicine physician automatically delineated the liver (using Auto-Contour ProtegeAI workflow) and manually delineated the tumour(s) on pre-therapeutic contrast-enhanced arterial T1-weighted MRI. Necrotic regions were included as part of the whole-lesion tumour VOI, consistent with MIRD-based absorbed dose calculation over the defined target volume [11]. These were independently transferred to the^99m^Tc-MAA SPECT/CT and the post-therapy ^90^Y PECT/CT using the Transfer Contours workflow. Contours were manually aligned using rigid transformation(translation, rotation and scaling) on each target dataset. Registration quality was systematically assessed by visual inspection of tumour, liver, and lung boundaries across at least two planes (axial, coronal, or sagittal), including assessment for possible interval tumour growth. In cases of substantial changes in lesion size or morphology, segmentation would have been based on the corresponding CT scan to avoid inaccuracies from lesion progression. No such cases occurred in this cohort. On this planning CT, additional contours of the lungs were created automatically using the Activity Planning workflow. All contours were subsequently transferred to the post-therapy ^90^Y PET/CT, and using the ^90^Y Dose Calculation workflow, to determine the actual delivered amount of energy at both tumours and non-affected tissues.

Dose–volume histograms (DVHs) for each lesion were generated from voxel-based absorbed dose maps exported from MIM SurePlan using the Local Deposition Method (LDM). Standard DVH parameters were extracted, including V100, V150, and V200, defined as the percentage of tumour volume receiving ≥ 100, ≥150, and ≥ 200 Gy, respectively. Hot-spot metrics included D2, D10, and Dmax, representing the minimum dose delivered to the hottest 2% and 10% of tumour volume and the maximum voxel dose. Cold-spot metrics included D90, D98, and Dmin, representing the minimum dose to 90% and 98% of tumour volume and the minimum voxel dose. D50 was defined as the median absorbed dose.

Intra-tumoral dose heterogeneity was quantified using the interquartile range (interquartile range (IQR= D25–D75), dose range (Dmax–Dmin), and coefficient of variation (CoV = SD/mean dose). For the non-tumoral liver, mean dose (NTLD) and dose heterogeneity (NTLD SD) were calculated from the same voxel-based dose distribution within the perfused non-tumoral liver volume, defined as the perfused liver minus all tumour VOIs.

### Clinical outcome measurement

Treatment-related toxicity was evaluated according to Common Terminology Criteria for Adverse Events (CTCAE) version 5.0 [12]. Adverse events were classified as acute if occurring within 3 months after SIRT and latent thereafter. Grade 3 or higher toxicity was defined as severe. Both laboratory toxicity and clinical symptomatic toxicity were systematically recorded. Toxicity was attributed to SIRT based on clinical review of treatment timing, baseline liver function, and changes from baseline laboratory values.

Imaging response was assessed on 4-month contrast-enhanced MRI using mRECIST criteria [13]. The four mRECIST categories, progressive disease (PD), stable disease (SD), partial response (PR), and complete response (CR), were treated as an ordered outcome (PD < SD < PR < CR) for ordinal regression analyses. All response and toxicity assessments were based on data obtained prior to any subsequent treatment.

To minimize partial-volume effects in post-therapy ^90^Y PET/CT-based dosimetry, lesions with a diameter < 20 mm were excluded [14]. For stratified analyses, lesions were classified into low-D50 (< 128 Gy) and high-D50 (≥ 128 Gy) groups based on the cohort median absorbed dose, allowing approximately balanced exploratory subgroups. Overall survival (OS) was defined as the time from the first SIRT to death or last clinical follow-up. In a sensitivity analysis, patients were censored at the time of orthotopic liver transplantation.

### Statistical analysis

All statistical analyses were performed using R (version 4.3.2). Continuous variables were reported as median (IQR) and categorical variables as counts and percentages. Group comparisons used the Wilcoxon rank-sum test and Kruskal-Wallis test with Dunn tests for pairwise comparisons. Associations between dose metrics and mRECIST response were evaluated using ordinal logistic regression adjusted for tumour volume. NTLD-toxicity associations were evaluated using logistic regression adjusted for age, BMI, liver volume, and lung dose. Adjusted predicted probabilities were plotted by varying the dose metric across its observed range, with continuous covariates fixed at their median values and categorical covariates at the reference category. Associations between tumour dose coverage and overall survival using Cox proportional hazards models, with exploratory sensitivity analyses adjusted separately for BCLC stage and Child-Pugh class. P values were adjusted for multiple comparisons using the Benjamini–Hochberg false discovery rate method. *P* < 0.05 was considered statistically significant.

## Results

### Demographics

A total of 45 patients with 68 target lesions treated with ^90^Y resin SIRT were included. The median tumour volume was 22.9 mL (10.2–71.6). Table 1 presents patient baseline characteristics. The median age was 71 years (63–77), with 77.8% male patients. Most patients had preserved liver function (86.7% Child–Pugh A) and were staged as Barcelona Clinic Liver Cancer (BCLC) A or B (84.5%) at baseline. The median injected activity was 1450 MBq (1200–2000). At the time of analysis, 37.8% of patients had died. Deaths were mainly related to disease progression, with 3 treatment-related deaths (liver failure, variceal bleeding, and radiation-induced liver disease). Median PFS was 8.2 months, while median OS was not reached (median follow-up 14.2 months). Grade ≥ 3 toxicity occurred in 40.0% of patients within 3 months after SIRT (acute phase) and in 33.3% beyond 3 months (latent phase).

**Table 1:** Baseline patient characteristics

Characteristic	All patients (*n* = 45)
Age, years	71 (63–77)
Sex, n (%)	
Male	35 (77.8)
Female	10 (22.2)
Child–Pugh class, n (%)	
A	39 (86.7)
B	6 (13.3)
BCLC stage, n (%)	
A	17 (37.8)
B	21 (46.7)
C	7 (15.6)
Indication for SIRT	
Solitary HCC	24 (53.3%)
Multifocal HCC	13 (28.9%)
Locally invasive HCC	2 (4.4%)
Extrahepatic disease/metastatic HCC	2 (4.4%)
Other	4 (8.9%)
Prior treatment, n (%)	
None	28 (62.2)
Ablation (RFA/MWA)	8 (17.8)
TACE	2 (4.4)
Surgery/OLT	3 (6.7)
Mixed therapy	4 (8.9)
Subsequent treatment, n (%)	
None	29 (64.4)
Ablation	5 (11.1)
Repeat SIRT	5 (11.1)
Systemic therapy	5 (11.1)
OLT	3 (6.7)
Resection	1 (2.2)
Albumin (g/L)	40 (38–42)
Bilirubin (µmol/L)	10 (8–16)
INR	1.1 (1.0–1.2)
Total tumour volume (mL)	206 (110–306)
Tumour-to-liver uptake ratio	6.8 (4.2–11.1)
Injected activity (MBq)	1450 (1200–2000)
PFS (months)	8.2 (4.6–16.4)
OS (months)	14.2 (9.1–24.3)
Any grade ≥ 3 toxicity, n (%)	
Acute	18 (40.0)
Latent	15 (33.3)
Laboratory grade ≥ 3 toxicity, n (%)	
Acute	6 (13.3)
Latent	5 (11.1)
Symptoms grade ≥ 3 toxicity, n (%)	
Acute	15 (33.3)
Latent	16 (35.6)

Abbreviations: *BCLC* Barcelona Clinic Liver Cancer, *INR* international normalized ratio, *MBq* megabecquerel, *MWA* microwave ablation, *OLT* orthotopic liver transplantation, *OS* overall survival, *PFS* progression-free survival, *RFA* radiofrequency ablation, *TACE* transarterial chemoembolization

### Tumour response

**Fig. 1:**
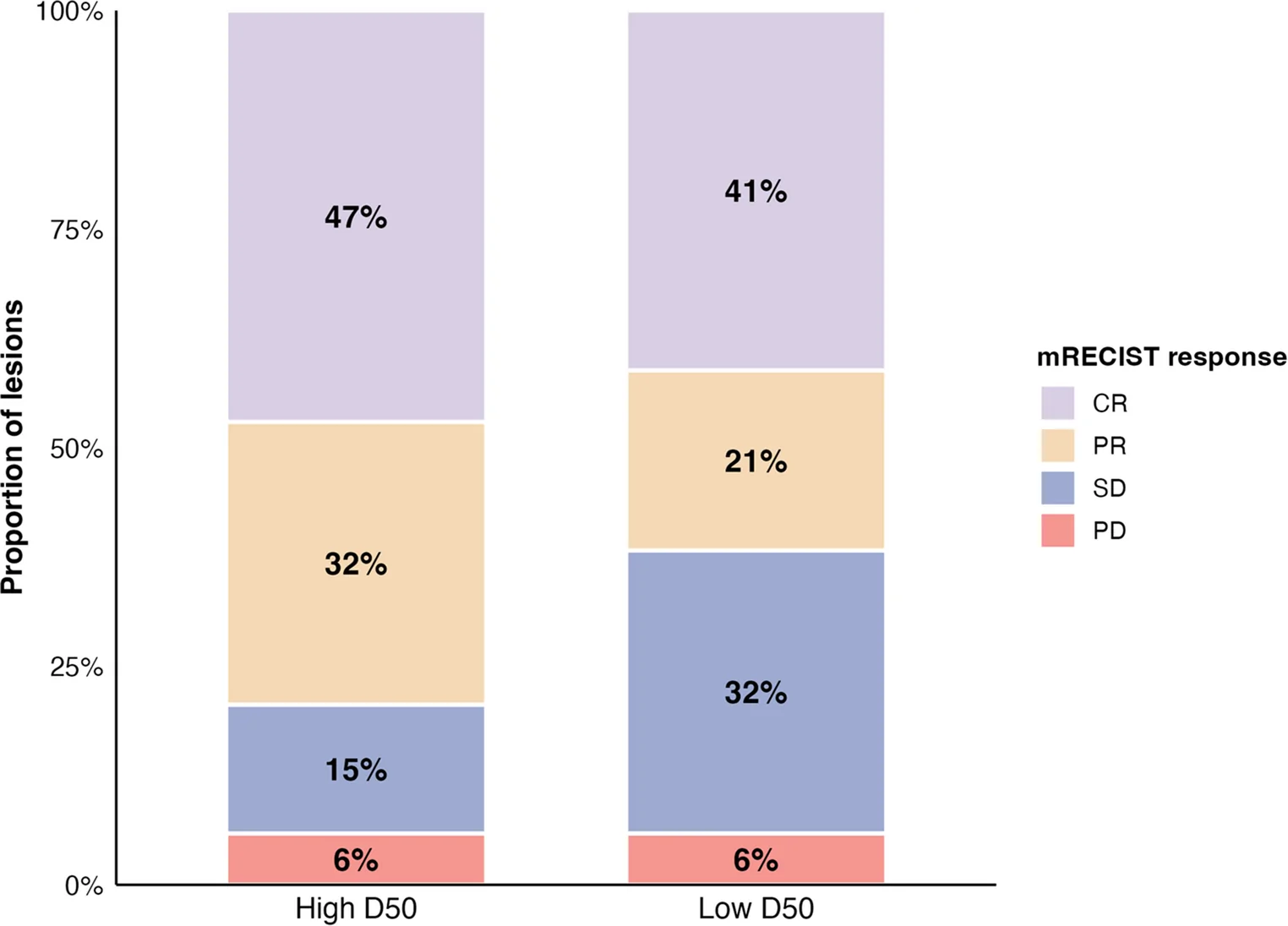
Distribution of mRECIST response categories in high-D50 and low-D50 groups

**Fig. 2:**
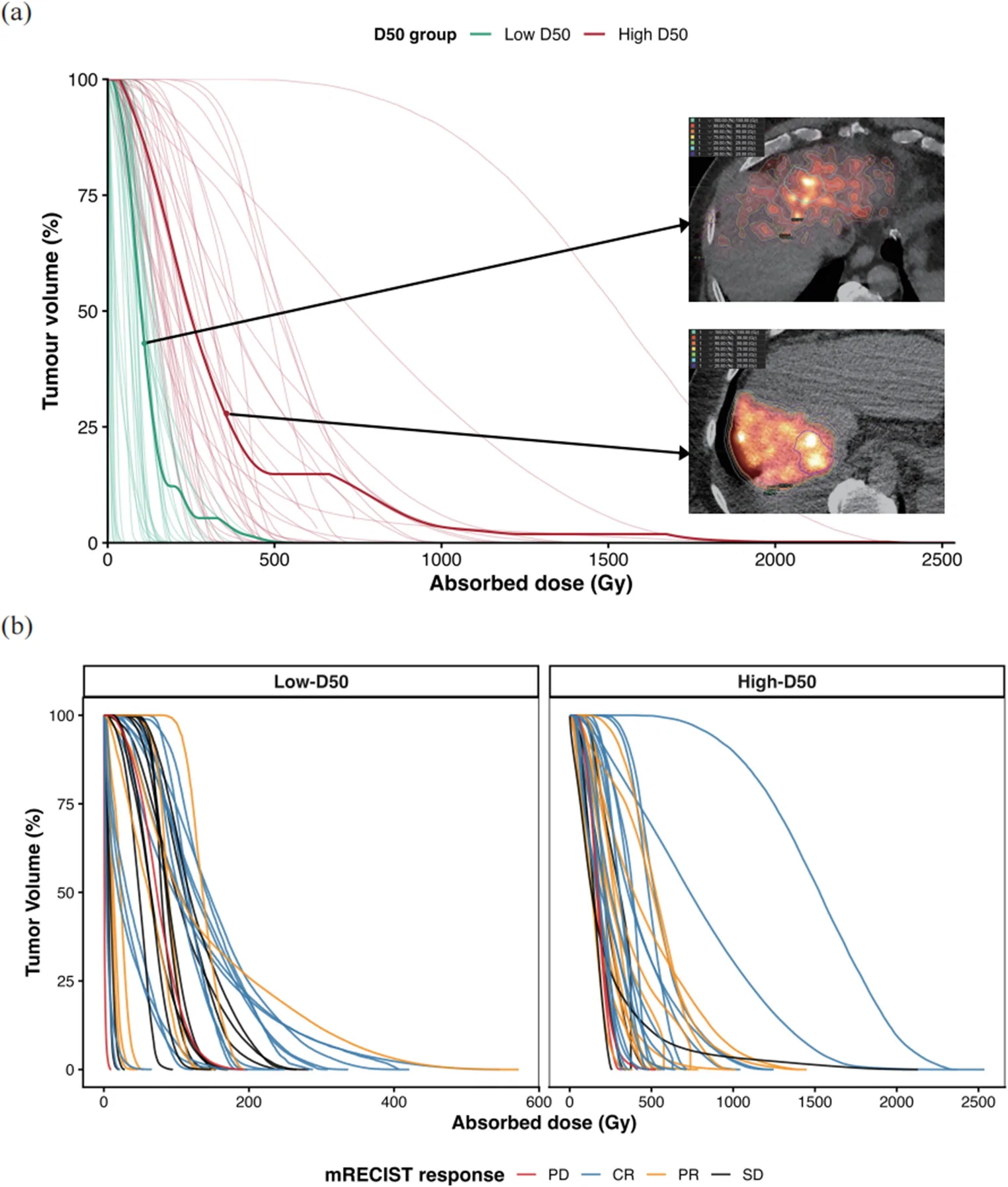
Representative dose distributions and cumulative dose-volume histograms. (**a**) Representative cumulative DVHs and post-therapy ^90^Y PET/CT dose maps for lesions in the low-D50 and high-D50 groups. The selected examples were the lesions with D50 values closest to the mean D50 within each subgroup. The low-D50 representative lesion had D50 = 73.1 Gy and PD, whereas the high-D50 representative lesion had D50 = 307 Gy and CR. (**b**) Cumulative DVHs of all lesions, stratified by D50 group and mRECIST response category

Of the 68 lesions, 30 (44.1%) achieved CR, 18 (26.5%) PR, 16 (23.5%) SD, and 4 (5.9%) PD. Lesions were stratified by D50 into high-D50 (≥ 128 Gy, *n* = 34) and low-D50 groups (< 128 Gy, *n* = 34). The distribution of mRECIST response categories did not differ significantly across groups (Wilcoxon rank-sum test, *p* = 0.331, Fig. 1). The cumulative DVHs of all lesions, stratified by D50 group and response category (Fig. 2).

### Dose heterogeneity and tumour response

**Fig. 3:**
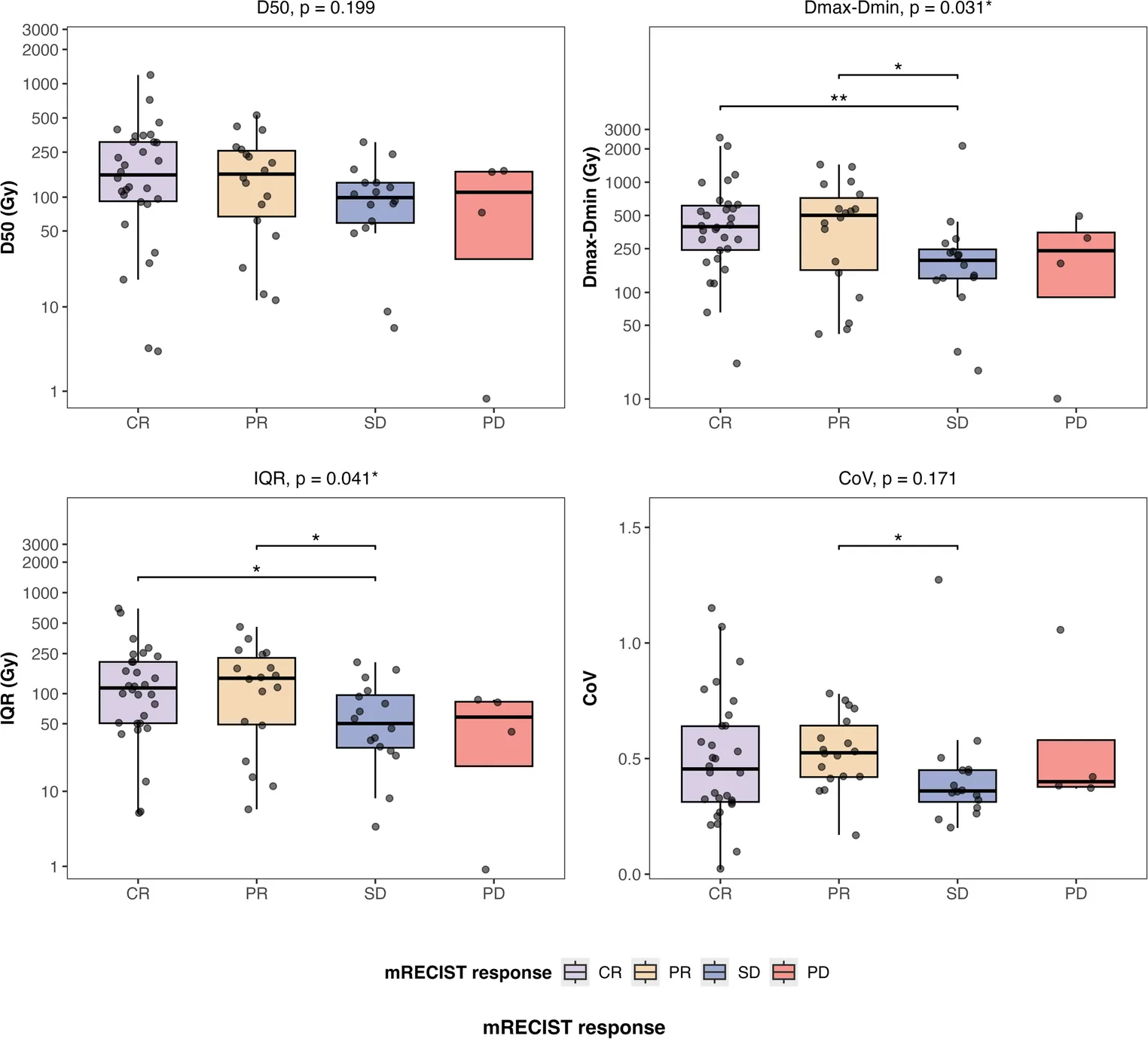
Distribution of tumour dose metrics across mRECIST response categories. Boxplots show D50, Dmax–Dmin, IQR, and CoV. Individual data points represent single lesions. Kruskal-Wallis test; asterisks indicate significant pairwise differences (Dunn test, *p < 0.05, **p < 0.01)

Figure 3 shows the distribution of tumour dose metrics across mRECIST response categories without dose stratification. Absolute heterogeneity metrics including Dmax–Dmin and IQR demonstrated significant differences across response categories (*p* = 0.031 and *p* = 0.041, respectively), with higher values in CR and PR lesions compared with SD. CoV did not show a significant association with response (*p* = 0.171). D50 did not differ significantly among response groups (Kruskal–Wallis test, *p* = 0.199), but was significantly higher in responder lesions (CR/PR) compared with non-responders (SD/PD) (Wilcoxon rank-sum test, *p* = 0.033; Online Resource 1). Two CR lesions with D50 values below 10 Gy were predominantly necrotic at baseline, while one notably high-D50 lesion (1193 Gy) occurred after highly selective microsphere delivery in a post-transplant patient.

The association between dose heterogeneity and response varied by absorbed dose level (Table 2). In low-D50 lesions, higher heterogeneity was associated with improved response. Range (OR 1.05 per 10 Gy, 95% CI 1.00–1.10, *p* = 0.048) and CoV (OR 1.34 per 0.1unit, 95% CI 1.00–1.80, *p* = 0.049) reached significance, with IQR showing a similar trend (OR 1.17 per 10 Gy, *p* = 0.085). In contrast, no heterogeneity metric was significantly associated with response in high-D50 lesions (all *p* > 0.05). A significant dose–heterogeneity interaction was observed for CoV (interaction *p* = 0.010).

**Table 2:** Association between intra-tumoral dose heterogeneity and mRECIST response stratified by median absorbed dose

Variable	D50 group	Adjusted OR (95% CI)	*P* value	Interaction *P*
Range (Gy)	High	1.02 (1.00–1.03)	0.055	0.109
	Low	1.05 (1.00–1.10)	0.048*	
IQR (Gy)	High	1.06 (0.99–1.13)	0.084	0.271
	Low	1.17 (0.98–1.41)	0.085	
CoV	High	0.88 (0.59–1.31)	0.535	0.010*
	Low	1.34 (1.00–1.80)	0.049*	

Adjusted OR from ordinal logistic regression models, using ordered mRECIST response categories (PD < SD < PR < CR), adjusted for tumour volume. ORs per 10-Gy (range, IQR) or per 0.1unit (CoV). Interaction P: test for effect modification by D50 group. *p < 0.05

After Benjamini-Hochberg correction, the subgroup-specific associations did not remain statistically significant; however, the interaction between CoV and D50 group remained significant (adjusted *p* = 0.030)

Abbreviations: *CI* confidence interval, *CoV* coefficient of variation, *D50* median absorbed dose, *IQR* interquartile range, *OR* odds ratio

In low-D50 lesions, hot-spot metrics D2 (OR 1.06 per 10 Gy, *p* = 0.060), D10 (OR 1.08, *p* = 0.065), and Dmax (OR 1.05, *p* = 0.061) demonstrated trends toward improved response, whereas no cold-spot metric reached significance (Table 3). In high-D50 lesions, cold-spot metrics D90 (OR 1.14 per 10 Gy, *p* = 0.032) and D98 (OR 1.13, *p* = 0.049) were significantly associated with better response, while hot- spot metrics showed no significant associations (Table 3). In high-D50 lesions, significant differences across response categories were observed for D2 (CR vs. PD, PD vs. PR), D10 (CR vs. PD), D90 (CR vs. SD), D98 (CR vs. PR), and minimum dose (CR vs. PR) (Table 4). No significant differences were observed in low-D50 lesions. An exploratory analysis of absolute underdosed tumour volume and a sensitivity analysis excluding lesions with visible necrotic components are presented in Online Resource 2.

**Table 3:** Dose–response associations stratified by D50

Region	Variable	Low D50	High D50
Hot spots (Gy)	D2	1.06 (1.00–1.12), *p* = 0.060 ·	1.01 (0.99–1.03), *p* = 0.164
	D10	1.08 (1.00–1.16), *p* = 0.065 ·	1.03 (1.00–1.06), *p* = 0.078 ·
	Max	1.05 (1.00–1.10), *p* = 0.061 ·	1.01 (0.99–1.02), *p* = 0.254
Cold spots (Gy)	D90	0.99 (0.77–1.26), *p* = 0.907	1.14 (1.01–1.28), *p* = 0.032 *
	D98	1.05 (0.79–1.39), *p* = 0.728	1.13 (1.00–1.29), *p* = 0.049 *
	Min	1.15 (0.78–1.70), *p* = 0.477	1.27 (0.97–1.66), *p* = 0.085 ·

ORs represent the change in odds of achieving a better mRECIST response per 10 Gy increase. * *p* < 0.05; · trend (*p* < 0.1)

These associations did not remain significant after Benjamini-Hochberg correction and are therefore presented as exploratory

Abbreviations: D2, D10, D90, and D98, minimum dose to the hottest 2%, 10% and to 90%, 98% of tumour volume, respectively; *D50* median absorbed dose, *Max* maximum voxel dose, *Min* minimum voxel dose, *mRECIST* modified Response Evaluation Criteria in Solid Tumours, *OR* odds ratio

**Table 4:** Pairwise comparisons across mRECIST response categories in low-D50 and high-D50 lesions (Dunn test)

Group	Region	Variable	Pairwise comparison	*P* value
Low D50		–	No significant pairwise differences	–
High D50	Hot spots (Gy)	D2	CR > PD	0.046 *
		D2	PR > PD	0.045 *
		D10	CR > PD	0.035 *
	Cold spots (Gy)	D90	CR > SD	0.044 *
		D98	CR > PR	0.026 *
		Min	CR > PR	0.016 *

*P* values are adjusted *p* values from Dunn’s post-hoc test. * *p* < 0.05

Abbreviations: *CR* complete response, *PR* partial response, *SD* stable disease, *PD* progressive disease, *D50* median absorbed dose, *mRECIST* modified Response Evaluation Criteria in Solid Tumours

### Non-tumour liver dose and toxicity

**Fig. 4:**
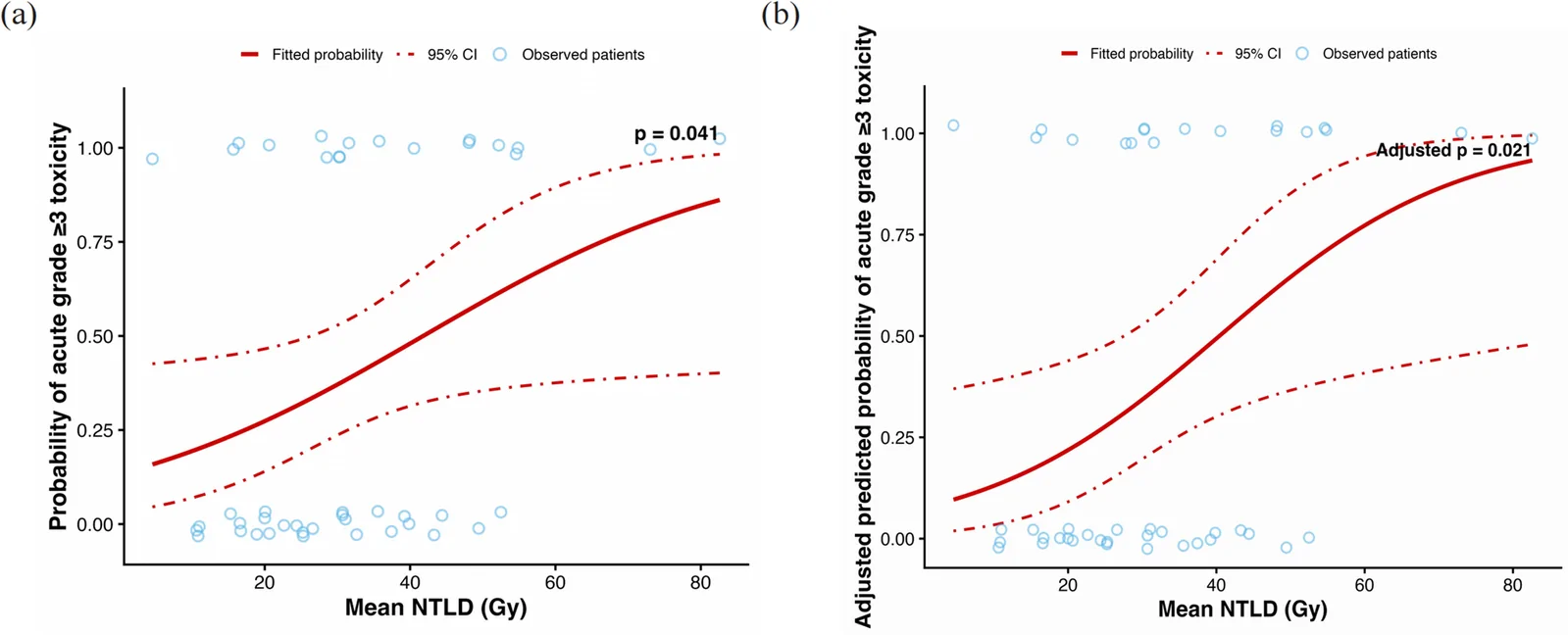
Association between mean NTLD and acute grade ≥ 3 toxicity. (**a**) Univariable logistic regression. (**b**) Multivariable logistic regression adjusted for age, BMI, non-tumour liver volume, and lung dose. Open circles indicate individual patient observations. Solid lines represent fitted logistic regression curves, and dot-dash lines indicate 95% confidence intervals

**Fig. 5:**
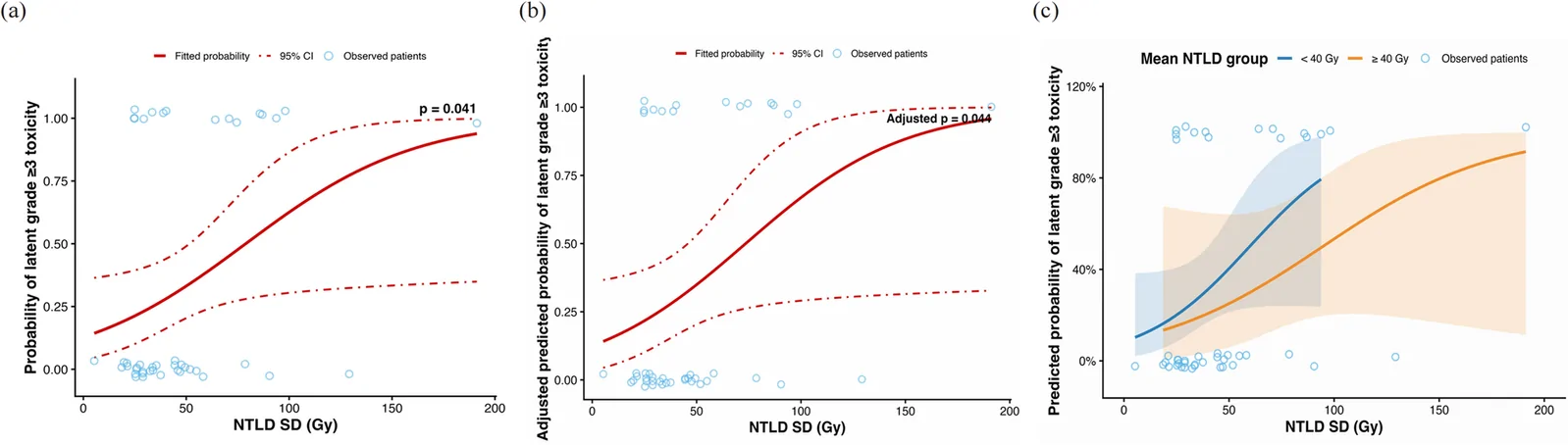
Association between NTLD SD and latent grade ≥ 3 toxicity. (**a**) Univariable logistic regression. (**b**) Multivariable logistic regression adjusted for age, BMI, and non-tumour liver volume. (**c**) Stratified analysis comparing patients with lower (< 40 Gy) and higher (≥ 40 Gy) mean NTLD. Open circles indicate individual patient observations. Solid lines represent fitted logistic regression curves, and dot-dash lines or shaded bands represent 95% confidence intervals

The median non-tumoral liver absorbed dose was 30.3 Gy (20.1–40.5), and the median NTLD SD was 37.6 Gy (26.0–58.2). Acute grade 3 or higher treatment-related toxicity occurred in 18 patients (40%), and latent severe toxicity in 15 patients (33%). Among the three grade 5 treatment-related toxicity cases, mean NTLD values ranged from 15.7 to 35.8 Gy and NTLD SD values from 24.8 to 29.4 Gy. Higher mean NTLD was associated with increased risk of acute toxicity (unadjusted OR 1.56 per 10 Gy, *p* = 0.041; adjusted OR 1.87 per 10 Gy, *p* = 0.021) (Fig. 4). The dose–toxicity relationship showed a continuous rise in toxicity probability with increasing NTLD, without evidence of a threshold effect. NTLD SD was associated with latent toxicity (unadjusted OR 1.02 per Gy, 95% CI 1.00–1.05, *p* = 0.041; adjusted OR 1.02 per Gy, 95% CI 1.00–1.05, *p* = 0.044). Stratified analysis showed that patients receiving lower mean NTLD (< 40 Gy) showed a stronger association (OR 1.04 per Gy, *p* = 0.077) compared with those receiving ≥ 40 Gy (OR 1.03 per Gy, *p* = 0.20) (Fig. 5).

### Tumour dose coverage and overall survival

**Fig. 6:**
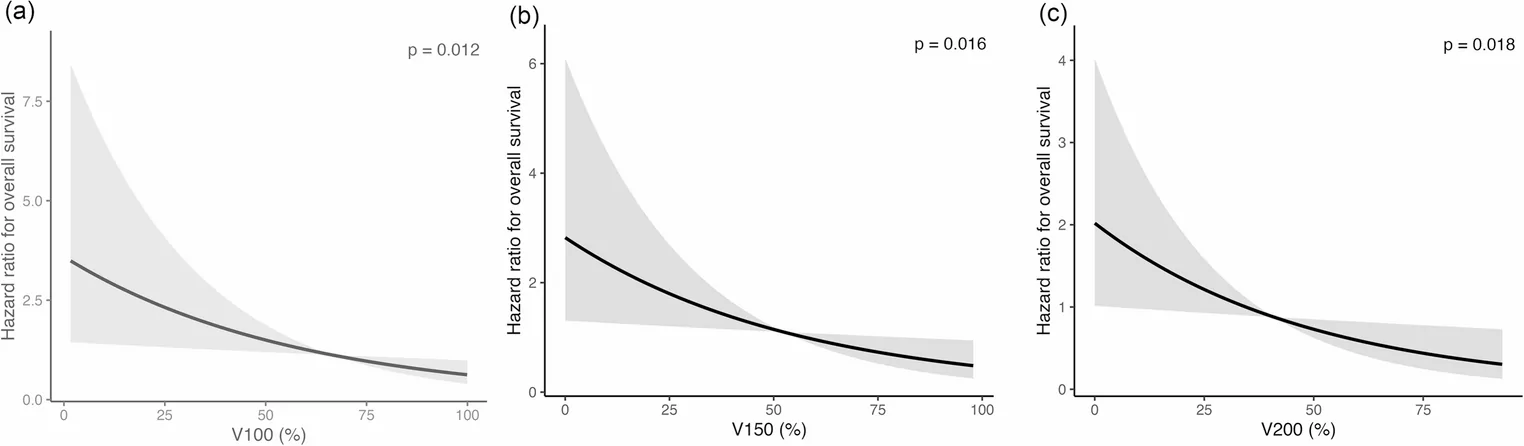
Association between tumour dose coverage and overall survival. Cox proportional hazards models showing HR curves for (**a**) V100 (% of tumour volume receiving ≥ 100 Gy), (**b**) V150 (% ≥150 Gy), and (**c**) V200 (% ≥200 Gy). Shaded areas represent 95% confidence intervals. HR > 1 indicates increased mortality risk

Higher tumour dose coverage was associated with better overall survival (Fig. 6). V100, V150, and V200 were associated with lower mortality risk. These associations remained significant in exploratory Cox sensitivity analyses adjusted separately for BCLC stage and Child-Pugh class. In a sensitivity analysis censoring patients at OLT, the association between tumour dose coverage and overall survival remained significant (*P* = 0.016–0.027; Online Resource 3). The HR curves crossed unity at approximately 66.5%, 52.4%, and 40.9%, respectively, indicating lower mortality risk above these coverage thresholds.

## Discussion

This study suggests that spatial dose distribution, rather than absorbed dose magnitude alone, may be an important determinant of outcome in HCC patients treated with ⁹⁰Y resin microsphere SIRT. Our findings suggest that the effect of dose heterogeneity on tumour response is dose-dependent, and non-tumoral liver dose heterogeneity may contribute to toxicity. These findings highlight the importance of spatial dosimetry characteristics for interpreting treatment response and toxicity after SIRT. Because all treatments used same-day calibrated resin microspheres, the observed dose heterogeneity likely reflects macroscopic vascular delivery patterns rather than microscopic inter-sphere dose [4].

Although higher D50 was associated with objective response, in line with previous reports in resin-treated HCC [5, 15], substantial overlap in D50 distributions across response categories indicates that D50 alone cannot fully explain treatment outcomes. DVH-based metrics capture heterogeneous microsphere distribution and have been associated with tumour response [16]. However, DVH-based dose alone may be insufficient to characterize intratumoral dose dispersion, partly because these metrics can be strongly inter-correlated due to the limited spatial resolution [17]. Absolute heterogeneity metrics such as range and IQR inherently scale with dose magnitude, and their associations with response in the unstratified analysis may partly reflect dose-level differences rather than spatial heterogeneity alone. Dose-stratified analyses were therefore performed to explore heterogeneity effects within lower and higher dose levels separately. A prior post-therapy ⁹⁰Y dosimetry study reported that DVH failed to predict lesion response, whereas a CoV cut-off of 0.79 had a positive predictive value in lesions receiving less than 50 Gy [18]. It suggested that incorporating heterogeneity metrics may offer additional predictive value, especially in low dose lesions. Our data showed similar results, with a higher CoV in the low-D50 (< 128 Gy) lesions associated with better response. Importantly, interaction analysis revealed a non-linear interaction between D50 level and CoV, with the beneficial effect of heterogeneity diminishing and potentially reversing at higher dose level. Given the limited subgroup size and borderline significance, this finding should be considered exploratory and requires confirmation in larger cohorts.

We explored whether intratumoral hot-spot and cold-spot dose extremes drive or restrict tumour response. Our results suggest that the dominant spatial determinant of response may differ according to overall dose level. In low dose lesions, hot spots showed consistent positive trends, suggesting that focal dose intensification may play a meaningful role in initiating tumour control when overall dose coverage is limited. This finding supports the concept that, in insufficiently dosed tumours, response may be driven by highly irradiated subregions rather than uniform dose delivery. This interpretation is supported by the discovery that Dmax, rather than mean absorbed dose, served as the best predictor of tumour response after ^90^Y-SIRT, and the predictive power of absorbed dose progressively decreased as the volume threshold increased from 30% to 70% indicates that response is primarily driven by the highest local microsphere concentration within hypervascularized tumour subregions rather than by dose coverage of the entire lesion [19]. These observations suggested that local regions of high microsphere concentration within hypervascularized tumour compartments may disproportionately contribute to treatment efficacy. Biological evidence further supports this mechanism, as high local radiation doses have been shown to induce vascular damage and immunogenic cell death, thereby amplifying tumour control beyond the directly irradiated volume [9, 20].

In contrast, once higher overall dose levels are achieved, residual underdosed subvolumes may present a key limitation to tumour control. Prior studies have demonstrated that the volume of tumour receiving < 100 Gy increased stepwise alongside worsening treatment response [7], consistent with tumour control probability principles whereby survival of clonogenic cells within inadequately treated subvolumes governs local outcome once sufficient overall dose is delivered [21]. In line with this framework, we observed that in high-D50 lesions, higher cold spots (D90, D98) were associated with superior response, indicating that minimization of low-dose regions may become increasingly relevant once adequate overall dose delivery is achieved. These metrics may thus serve as complementary surrogates of cold-spot burden in adequately dosed lesions. This concept may also apply to radiation segmentectomy with either glass or resin microspheres [22], where ablative dose delivery depends on both high absorbed dose and adequate target coverage. Although resin and glass microspheres differ in particle number and specific activity, post-therapy PET/CT captures macroscopic vascular-level dose heterogeneity within the tissue penetration range of ⁹⁰Y β-particles (mean range, 2–4 mm [1]), which remains clinically relevant for both platforms.

In addition to tumour dose, this study further evaluated the relationship between non-tumoral liver dose characteristics and treatment-related toxicity after ^90^Y SIRT. Mean NTLD was significantly associated with acute grade ≥3 toxicity, consistent with prior reports, although no clear dose threshold was found [3, 23]. This suggests that acute toxicity risk may increase progressively with dose, even below commonly cited safety limits [24, 25]. Spatial dose dispersion in non-tumoral liver, quantified by NTLD SD, showed an association with latent severe toxicity, particularly among patients receiving lower mean NTLD (< 40 Gy). This finding suggests that spatial dose heterogeneity may offer additional insight into delayed toxicity risk [18]. However, NTLD mean and NTLD SD are related metrics and may reflect partly overlapping aspects of non-tumoral liver dose exposure. Therefore, the apparent distinction between mean dose-related acute toxicity and heterogeneity-related late toxicity remains exploratory and requires validation in larger cohorts.

Prior studies highlighted the clinical relevance of dose coverage metrics, with V100 and underdosed tumour volume being associated with tumour response and survival [7, 26]. In line with this, we found that absolute underdosed tumour volume was associated with complete response in univariable analysis, but this association weakened after adjustment for tumour volume, suggesting that percentage-based coverage may capture response-related information beyond lesion size alone. Extending these observations, we quantified dose coverage at escalating dose levels and found that coverage of at least 66.5% of tumour volume at 100 Gy, 52.4% at 150 Gy, and 40.9% at 200 Gy was associated with improved overall survival in our cohort, supporting the importance of adequate volumetric dose coverage as a complement to spatial heterogeneity metrics. These coverage metrics may also provide information complementary to D50, as D50 summarizes the central tumour dose, whereas V100, V150, and V200 reflect the extent of tumour volume receiving adequate dose and may be more sensitive to residual underdosed regions. This is consistent with tumour response and overall survival being related but distinct endpoints, with survival additionally influenced by tumour burden, liver function, subsequent treatments, and overall disease course [2].

This study has some limitations. The retrospective design limits causal inference, and unmeasured confounding may remain. The sample size, although substantial for voxel-based dosimetry, constrained subgroup analyses by tumour size and baseline liver function. Because VOI propagation was based on rigid registration, small residual mismatches between baseline and post-therapy imaging may have remained despite visual verification, particularly for boundary-sensitive DVH metrics such as D98 and D2. Whole-lesion dosimetry did not separately assess viable enhancing tumour coverage. Baseline imaging was performed within 12 weeks before treatment, reflecting clinical workflow constraints, and interval tumour changes may have affected contour accuracy in individual cases [27]. Inter-observer variability in perfused liver volume was low in a small subset of patients, but larger reproducibility assessments are needed to fully characterize contouring-related uncertainty. Response assessment at approximately 4 months may also underestimate delayed tumour response [28], and incomplete longer-term follow-up limited further dose-response assessment. This was a single-centre experience with resin microspheres and may not be applicable to glass microspheres, which have different physical properties and dose distributions. Dose stratification used a D50-based cut-point of 128 Gy, this data-driven value may not represent a biological threshold, and alternative stratification approaches could yield different interaction patterns. Post-therapy ^90^Y PET/CT performed within 24 h after SIRT may not capture late redistribution or biological modulation of dose effect. Finally, our analyses focused on delivered physical dose, estimating biologically effective dose would require dose-rate and radiobiological modelling.

These findings warrant prospective validation in larger cohorts and may inform future efforts to integrate spatial dosimetry into treatment planning and toxicity risk assessment.

## Conclusion

Dose heterogeneity effects in ^90^Y resin SIRT appear dose dependent. In lower-dose lesions, treatment response was more strongly related to focal high-dose regions, whereas in higher-dose lesions, adequate coverage of underdosed subvolumes became more important. For toxicity, mean non-tumoral liver dose was associated with acute severe events, while non-tumoral liver dose heterogeneity was associated with latent severe toxicity, supporting the integration of spatial dosimetry into individualized treatment planning.

## Supplementary Information

Below is the link to the electronic supplementary material.

Supplementary Material 1 Online Resource 1. Additional statistical results for tumour dose and response analyses.

Supplementary Material 2 Online Resource 2. Exploratory analysis of absolute underdosed tumour volume and complete response.

Supplementary Material 3 Online Resource 3. Sensitivity analysis of overall survival after censoring patients at orthotopic liver transplantation.

## Data Availability

The datasets generated during and analysed during the current study are not publicly available due to patient privacy regulations but are available from the corresponding author on reasonable request.
