# Supplementary material for: Effects of dose heterogeneity on response and toxicity after 90Y-SIRT for HCC
Source: Eur J Nucl Med Mol Imaging. 2026 Jul 14;53(12):7071–81. doi: 10.1007/s00259-026-08064-1 (PMC13633298; doi:10.1007/s00259-026-08064-1)
Supplement: Supplementary file 1 [file 259_2026_8064_MOESM2_ESM.docx]

Supplementary Information

Article title: Effects of Dose Heterogeneity on Response and Toxicity After ^90Y-SIRT for HCC

Journal name: European Journal of Nuclear Medicine and Molecular Imaging

Authors: Huan Xi, Xinlin Zheng, Andor W.J.M. Glaudemans, Joyce van Sluis, Simeon J.S. Ruiter, Robbert J. de Haas, G. Matthijs Kater, Oleksandra V. Ivashchenko, Walter Noordzij

Affiliations:

1. Department of Nuclear Medicine and Molecular Imaging, University of Groningen, University Medical Centre Groningen, Groningen, The Netherlands

2. Department of Hepato-Pancreato-Biliary Surgery and Liver Transplantation, University of Groningen, University Medical Centre Groningen, Groningen, The Netherlands

3. Department of Radiology, University of Groningen, University Medical Centre Groningen, Groningen, The Netherlands

Corresponding author: Huan Xi

E-mail: h.xi@umcg.nl

Online Resource 2. Exploratory analysis of absolute underdosed tumour volume and sensitivity analysis excluding lesions with visible necrotic components

Table S2.1. Exploratory analysis of absolute underdosed tumour volume and complete response

| **Metric** | **Complete response** | **Non-complete response** | **P value** | **Adjusted OR for CR** | **95% CI** | **P value** |
| --- | --- | --- | --- | --- | --- | --- |
| Underdosed volume <100 Gy, mL | 2.25 (0.46–6.20) | 11.79 (3.82–35.97) | 0.00020 | 0.81* | 0.58–1.14 | 0.229 |
| Underdosed volume <150 Gy, mL | 4.36 (1.38–11.82) | 20.72 (6.20–44.51) | 0.000079 | 0.75* | 0.53–1.07 | 0.115 |
| Underdosed volume <200 Gy, mL | 5.36 (2.02–14.75) | 23.38 (8.35–58.86) | 0.000069 | 0.71* | 0.50–1.02 | 0.066 |
| V100, per 10% increase | — | — | — | 1.17† | 1.01–1.36 | 0.035 |
| V150, per 10% increase | — | — | — | 1.21† | 1.04–1.41 | 0.016 |
| V200, per 10% increase | — | — | — | 1.26† | 1.06–1.50 |  |

Values are median (IQR) unless otherwise indicated. P values for complete response versus non-complete response were calculated using the Wilcoxon rank-sum test.

*Adjusted for tumour volume; OR per 10 mL increase in absolute underdosed tumour volume.

†Adjusted for tumour volume; OR per 10% increase in percentage-based coverage metric.

Abbreviations: CI, confidence interval; CR, complete response; OR, odds ratio.

Table S2.2. Sensitivity analysis excluding lesions with visible necrotic components

| **Dose coverage metric** | Primary analysis, all lesions (n = 68) | Sensitivity analysis, excluding necrotic lesions (n = 65) |
| --- | --- | --- |
| V100, per 10% increase | OR 1.12 (95% CI: 1.00–1.27), p = 0.061 | OR 1.14 (95% CI: 1.01–1.30), p = 0.039 |
| V150, per 10% increase | OR 1.16 (95% CI: 1.03–1.32), p = 0.020 | OR 1.18 (95% CI: 1.04–1.35), p = 0.013 |
| V200, per 10% increase | OR 1.21 (95% CI: 1.06–1.40), p = 0.007 | OR 1.24 (95% CI: 1.07–1.44), p = 0.005 |

Values are adjusted odds ratios from ordinal logistic regression using ordered mRECIST response categories (PD < SD < PR < CR), adjusted for tumour volume. Three lesions with visible necrotic components within the baseline tumour VOI were excluded in the sensitivity analysis.

Abbreviations: CI, confidence interval; CR, complete response; mRECIST, modified Response Evaluation Criteria in Solid Tumours; OR, odds ratio.
